# Interfacial Polarization and Charge Transfer in Al_13_ Superatom–Organic Interfaces: Role of Frontier Orbital Overlap

**DOI:** 10.1021/acsomega.6c05879

**Published:** 2026-10-06

**Authors:** Meliton R. Chiong, Atsushi Nakajima, Yoshitada Morikawa

**Affiliations:** † Department of Precision Engineering, Graduate School of Engineering, The University of Osaka, 2-1 Yamadaoka, Suita, Osaka 565-0871, Japan; ‡ Department of Chemistry, Faculty of Science and Technology, 12869 Keio University, 3-14-1 Hiyoshi, Kohoku-ku, Yokohama 223-8522, Japan; § Innovative Catalysis Science Division, Institute of Open and Transdisciplinary Research Initiatives (ICS-OTRI), The University of Osaka, Suita, Osaka 565-0871, Japan; ∥ Research Center for Precision Engineering, Graduate School of Engineering, The University of Osaka, 2-1 Yamadaoka, Suita, Osaka 565-0871, Japan

## Abstract

Superatoms (SAs) have emerged as atomically precise building blocks for functional materials; however, their interfacial electronic interactions with organic substrates remain poorly understood, particularly in the absence of ligand protection. While most reported SAs are stabilized by ligands, ligand-free SAs provide a unique platform to probe intrinsic interfacial behavior. In particular, X-ray photoelectron spectroscopy (XPS) core-level shifts at SA-organic interfaces are interpreted as signatures of charge transfer, although the contributions of interfacial polarization remain unclear. Here, we present a comparative density functional theory (DFT) study of a ligand-free Al_13_ SA adsorbed on two π-conjugated substrates: hexa-*peri*-benzocoronene (HBC) and C_60_. Despite their comparable sizes and extended π systems, these substrates exhibit fundamentally distinct molecular interaction regimes. For Al_13_–HBC, minimal charge transfer from HBC to Al_13_ occurs and the interaction is dominated by dispersion and polarization, yet computed core-level shifts are consistent with an electron-enriched SA. In contrast, Al_13_–C_60_ shows substantial electron transfer from Al_13_ to C_60_, accompanied by covalent bond formation and pronounced electronic reconstruction. Real-space, dipole moment, and charge-partitioning analyses collectively reveal that core-level shifts can arise predominantly from interfacial polarization, even in the absence of substantial integer electron transfer. Projected density of states analysis further shows that charge transfer is enabled when favorable energy alignment is coupled with symmetry-allowed frontier orbital overlap. Environmental polarization and peripheral chemical substitution of HBC induce only minor changes, supporting that polarization alone is insufficient to drive electron transfer. These results establish that both energetic and symmetry constraints determine charge transfer at SA–organic interfaces, and provide design principles for superatomic hybrid materials.

## Graphical abstract

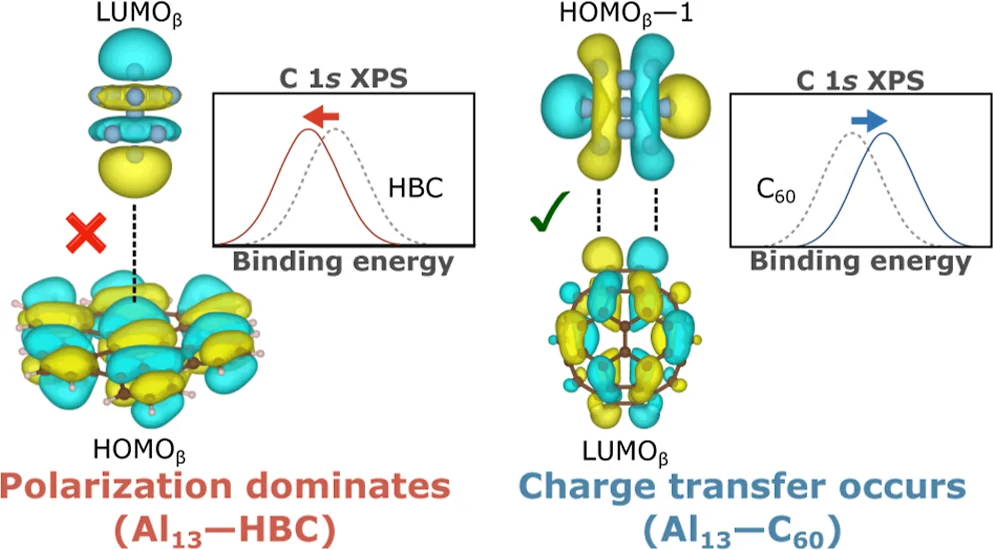

## Introduction

Superatoms (SAs) are an emerging class of clusters whose electronic states mimic the shell structure of individual atoms,^1,2^ offering a bottom-up approach to designing functional materials.^3–5^ While ligand-protected SAs have extensively been studied from the viewpoint of stabilizing cluster cores and enabling their chemical functionalization with ligands,^6–14^ a ligand-free framework is required to elucidate the intrinsic electronic structure of the superatomic core and its interaction with external materials. Bare SAs thus provide a direct platform to probe superatomic properties at interfaces without extrinsic perturbations. Among these, the icosahedral aluminum 13-mer, Al_13_, cluster is a prominent “halogen mimic” SA due to its high experimental electron affinity (EA) of 3.62 eV.^15,16^ When Al_13_ accepts an electron to become Al_13_^–^, it achieves a closed-shell electronic configuration which is exceptionally stable under the jellium model.^17,18^ While the Al_13_ cluster itself is superatomic, its “magic” stability is most clearly manifested in the anionic state.

Preserving the superatomic character of Al_13_ upon deposition on surfaces remains a challenge. Structural deformation, aggregation, and substrate-induced charge transfer can perturb its electronic structure and compromise its stability.^19^ In addition, Al-based systems are highly susceptible to oxidation, further complicating their integration into functional materials.^20,21^ As a result, considerable effort has been devoted to controlling the molecular environment of SAs, for example through ligand coordination and external fields, to modulate their electronic structure and reactivity.^22–29^ These approaches highlight the importance of the surrounding molecular environment in controlling superatomic properties.

Recent experimental works have provided important insight into the behavior of size-selected clusters on surfaces,^30,31^ including Al_13_ SAs deposited on organic substrates with distinct electronic characteristics.^32–34^ In studies of Al_13_ SAs, Al_13_ was soft-landed onto hexa-*tert*-butyl-hexa-*peri*-benzocoronene (HB-HBC), a p-type polycyclic aromatic hydrocarbon derivative, and C_60_, a well-known n-type electron acceptor. X-ray photoelectron spectroscopy (XPS) measurements revealed opposite trends in core-level shifts: Al_13_ on C_60_ exhibited cationic character and high susceptibility to oxidation, whereas Al_13_ on HB-HBC appeared relatively resistant to oxidation and was interpreted as reduced into superatomic Al_13_^–^ anion. The direction of charge transfer, anionic shifts on the p-type substrate and cationic shifts on the n-type substrate, suggested that the molecular environment dictates the electronic state of the Al_13_ SA.

However, XPS core-level shifts alone cannot unambiguously distinguish between integer charge transfer and interfacial polarization.^35^ Thus, it remains unclear whether Al_13_ on HB-HBC is fully reduced to the superatomic Al_13_^–^ anion, or instead remains largely neutral but polarized. This distinction is critical, as polarization-driven interactions and integer charge transfer lead to fundamentally different electronic and chemical behavior.

In this work, we employ DFT to examine the electronic structure of Al_13_ on HBC and C_60_ and to clarify the origin of the observed core-level shifts. While Al_13_–C_60_ exhibits substantial charge transfer with covalent bond formation, Al_13_–HBC is instead dominated by London dispersion forces with minimal net charge transfer. Nevertheless, interfacial polarization in Al_13_–HBC produces core-level shifts consistent with an electron-enriched SA, providing an alternative explanation without requiring full reduction to Al_13_^–^.

This contrast arises from differences in frontier orbital coupling. In Al_13_–HBC, a mismatch between the σ-type nodal structure of the Al_13_ lowest unoccupied molecular orbital of the beta channel (LUMO_β_) and the π-type HBC highest occupied molecular orbital (HOMO) leads to weak orbital overlap, suppressing electron transfer despite favorable energetics. In contrast, symmetry-compatible orbitals in Al_13_–C_60_ enable strong hybridization and efficient electron transfer.

These results indicate that interfacial polarization is intrinsic to SA–molecular interactions, whereas charge transfer is governed by the energetic and symmetry compatibility between the frontier orbitals, providing mechanistic insights for SA–organic interfaces. The present study focuses on the fundamental electronic interaction between a single Al_13_ cluster and a single molecular substrate. Although experimental thin-film systems may involve multiple neighboring molecules and may present collective interfacial effects, isolating the elementary SA–molecular interaction provides a direct means to identify the electronic factors governing charge transfer. In realistic interfaces, many-body polarization and cooperative interactions may modify the magnitude of charge redistribution. However, the requirement for efficient charge transfer is expected to remain applicable to individual SA–molecular interactions.

## Computational Details

All calculations were performed using density functional theory (DFT) as implemented in Gaussian09.^36^ The ground state structures of Al_13_–HBC and Al_13_–C_60_ are systematically identified using the spin-unrestricted UPBE0 hybrid functional^37^ with Grimme’s D3 dispersion correction^38^ and the basis set of 6-311+G­(d,p). Vibrational frequency analyses were carried out at the same level of theory to confirm that the optimized geometries correspond to true minima on the potential energy surface.

In the experimental studies, Al_13_ is deposited on HB-HBC. In the present work, we employ the unsubstituted HBC molecule as a model system, as the peripheral *tert*-butyl groups are not expected to significantly alter the electronic structure of the π-conjugated core. Consequently, the frontier orbital characteristics relevant to interfacial interaction with Al_13_ are expected to be well represented by HBC.

Wave function analyses were performed using Multiwfn,^39^ and visualization was carried out using Avogadro^40^ and VESTA.^41^ For the calculations of projected density of states (PDOS) and overlap integrals, diffuse functions were removed to avoid artificial long-range basis set mixing between fragments. Accordingly, the 6-311G­(d,p) basis set was employed for these analyses to ensure a well-defined orbital projection.

Upon adsorption, the basis functions of one fragment artificially stabilize the other as the fragments approach each other. This basis set superposition error (BSSE) leads to an overestimation of adsorption energies. To correct this effect, the counterpoise (CP) method was applied.^42^

## Results and Discussion

### Adsorption Structures and Energetics

The ground-state adsorption structures of Al_13_ on HBC and on C_60_ were identified through a systematic exploration of symmetry-distinct adsorption sites and approach geometries. For HBC (Figure 1a), geometry optimization was initially performed assuming an idealized planar (*D*_6*h*_) structure. This configuration exhibited six imaginary vibrational frequencies, indicating a saddle point rather than a true minimum. The ground-state structure of HBC was therefore obtained by successive reoptimizations along the imaginary modes until all vibrational frequencies became real. The resulting structure is slightly twisted and asymmetric (*C*_1_), while remaining nearly planar.

**Figure 1:**
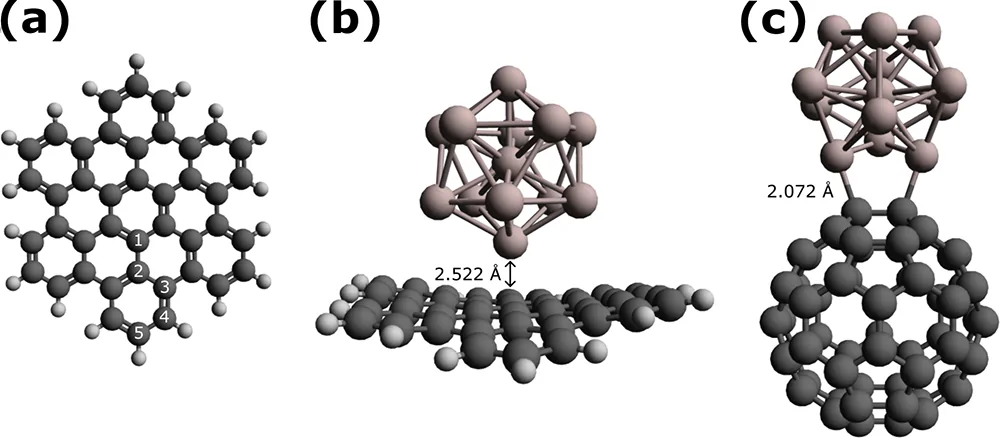
(a) The five different adsorption sites of Al_13_ on HBC. Ground-state structures of (b) Al_13_–HBC and (c) Al_13_–C_60_.

The optimized ground-state Al_13_ cluster (*D*_3*d*_) was then adsorbed onto HBC. Multiple initial adsorption orientations, including top-on, side-on, and face-on configurations, were considered; all structural relaxations led to a top-on geometry, as shown in Figure 1b. Adsorption at distinct carbon sites on HBC was further examined by considering the five inequivalent sites labeled 1 to 5 (Figure 1a). Among these, site 1 (innermost carbon) yielded the most stable configuration with an adsorption energy of −0.865 eV, whereas site 2 was least favorable (−0.754 eV). Initial placements at sites 3 and 5 relaxed to the same structure as site 4, resulting in identical adsorption energies (−0.814 eV). These results confirm that the ground-state structure of Al_13_–HBC corresponds to a top-on Al_13_ cluster above a carbon atom of the central ring, with an Al–C distance of 2.522 Å. The ground-state structure and the two higher-energy isomers obtained from the structural search are provided in the Supporting Information (Figure S1).

The ground-state structure of Al_13_–C_60_ was determined following an analogous procedure (Figure 1c). Isolated C_60_ adopts the *I*_*h*_ point group symmetry and provides high-symmetry adsorption sites: hexagonal face, pentagonal face, 5–6 bridge, 6–6 bridge, and atop a carbon. For each site, top-on, side-on, and face-on orientations of Al_13_ were examined, yielding 15 candidate adsorption geometries. In addition, adsorption of the capped Al_13_^+^ structure was also considered to account for possible charge transfer. The lowest-energy structure corresponds to side-on adsorption of Al_13_ at the 6–6 bridge site of C_60_ with *C*_2*v*_ symmetry, featuring an Al–C bond length of 2.072 Å. The ground-state structure and nine representative isomers identified in the structural search are provided in the Supporting Information (Figure S2).

Table 1 summarizes the counterpoise (CP)-corrected adsorption, complexation, and distortion energies of Al_13_–HBC and Al_13_–C_60_. The adsorption energy (Δ*E*_ads_^CP^) is defined as the energy difference between the isolated fragments and the resulting complex, the complexation energy (Δ*E*_complex_^CP^) as the instantaneous attractive energy between fragments at the final complex geometry, and the distortion energy, (Δ*E*_distort_) as the energetic cost of deforming the isolated fragments from their equilibrium geometries to those in the complex.

**Table 1:** Counterpoise (CP)-Corrected Adsorption (Δ*E*_ads_^CP^), Complexation (Δ*E*_complex_^CP^), and Distortion (Δ*E*_distort_) Energies for Al_13_–HBC and Al_13_–C_60_ (eV)^a^ ^,^ ^b^ ^,^ ^c^

system	Δ*E*_ads_^CP^ ^a^	Δ*E*complex^CP^ ^b^	Δ*E*_distort_ ^c^
Al_13_–HBC	–0.687	–0.881	0.193
Al_13_–C_60_	–1.204	–2.676	1.472

a Δ*E*_ads_^CP^ = *E*_*AB*_(AB_basis_)– [*E*_*A*_,relaxed­(*A*_basis_) + *E*_*B,*_relaxed­(*B*_basis_)] + BSSE = Δ*E*_ads_ + BSSE, where BSSE = [*E*_A_(*A*_basis_)+*E*_*B*_(*B*_basis_)] – [*E*_A_(*AB*_basis_) + *E*_*B*_(*AB*_basis_)].

b Δ*E*_complex_^CP^ = *E*_*AB*_(*AB*_basis_) – [*E*_A_(*A*_basis_) + *E*_*B*_(*B*_basis_)] + BSSE = Δ*E*_complex_ + BSSE.

c Δ*E*_distort_ = Δ*E*_binding_^CP^ – Δ*E*_complex_^CP^.

Al_13_–HBC exhibits substantially weaker adsorption (−0.687 eV) than Al_13_–C_60_ (−1.204 eV). In Al_13_–HBC, both the complexation and distortion energies are small, indicating adsorption without significant structural rearrangement of either fragment. This energetic signature is characteristic of physisorption dominated by weak dispersive interactions.

In contrast, Al_13_–C_60_ displays a large complexation energy accompanied by a substantial distortion penalty. The large distortion energy reflects significant reconstruction upon adsorption, which is energetically compensated by the strong stabilizing interaction between Al_13_ and C_60_. This behavior is indicative of chemisorption involving electronic reorganization and bond formation. While this energetic analysis distinguishes the two adsorption regimes, a detailed electronic structure analysis is required to elucidate the fundamental origin of this contrast.

### Electronic Structure and Bonding Analysis

#### Real-Space Analysis of Interactions

To directly assess the electronic nature of the Al_13_–substrate interaction, we first examine real-space descriptors of electron localization and charge redistribution. The electron localization function (ELF) provides a measure of electron pairing ranging from 0 (fully delocalized) to 1 (fully localized).^43^ Figure 2a shows three-dimensional ELF isosurfaces (ELF = 0.8) for Al_13_–HBC and Al_13_–C_60_. In Al_13_–HBC, no ELF localization is observed at the interface between the cluster and the substrate, indicating the absence of shared electron density. In contrast, Al_13_–C_60_ exhibits pronounced electron localization at the Al–C contact, providing direct evidence of an interfragment covalent bond.

**Figure 2:**
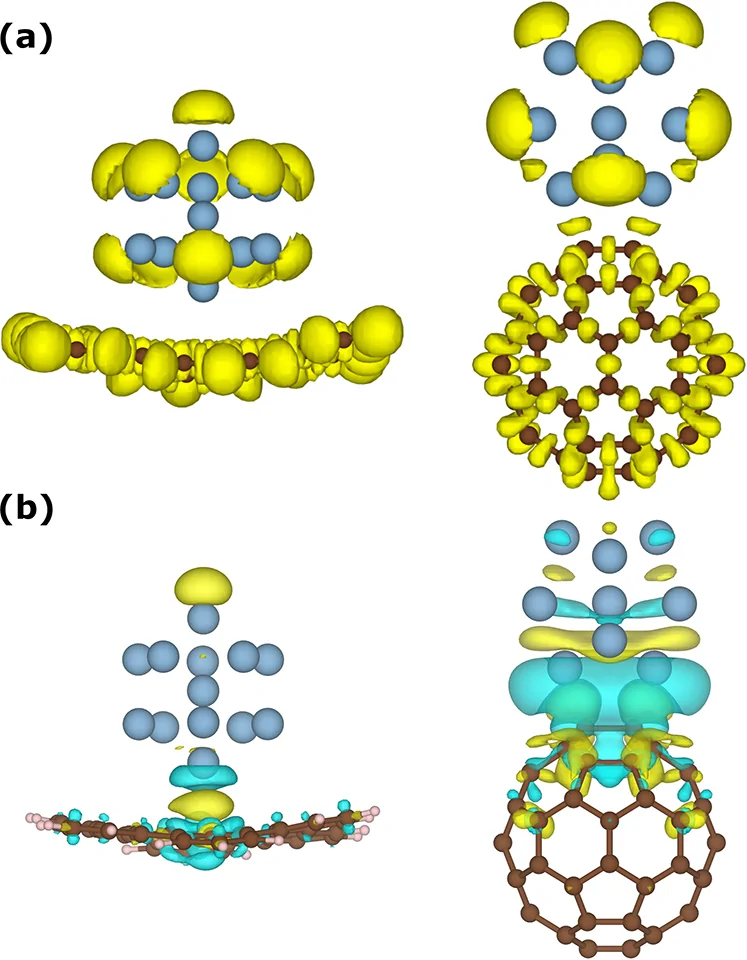
(a) Electron localization function (ELF) and (b) charge density difference (CDD) plots of Al_13_–HBC and Al_13_–C_60_. Yellow and cyan regions of CDD show the accumulation and depletion of electron density. The ELF plots are at isovalue of 0.8, while the CDD plots have isovalue of 0.001.

This distinction is further clarified by the charge density difference (CDD) upon complex formation, shown in Figure 2b, where regions of charge accumulation and depletion are depicted in yellow and cyan, respectively. For Al_13_–HBC, the CDD displays alternating accumulation and depletion across the two fragments, characteristic of mutual polarization consistent with London dispersion interactions.^44^ In contrast, Al_13_–C_60_ shows localized charge accumulation between Al and C atoms at the interface, accompanied by depletion from adjacent regions, reflecting directional electron sharing associated with covalent bonding. Together, the ELF and CDD analyses distinguish physisorption in Al_13_–HBC from chemisorption in Al_13_–C_60_.

Having established the absence of covalent bonding in Al_13_–HBC, we next analyze the noncovalent interactions responsible for stabilizing this complex. The reduced density gradient (RDG) was examined as a function of sign­(λ_2_)­ρ, where λ_2_ is the second eigenvalue of the electron density Hessian.^45^ The resulting two-dimensional scatter plots and three-dimensional RDG isosurfaces (RDG = 0.05) are shown in Figure S1. Peaks in the low-density, low-RDG region identify noncovalent interactions. The quantity sign­(λ_2_)*ρ* distinguishes interaction types and is color coded accordingly: blue indicates strong attractive interactions, green weak van der Waals interactions, and red steric repulsion. In Al_13_–HBC, a green RDG region appears at the interface, confirming that weak dispersion forces of van der Waals interactions dominate in the absence of covalent bonding. In Al_13_–C_60_, similar RDG features are present but oriented perpendicular to the Al–C bonds, indicating a secondary stabilizing role. In both complexes, red RDG regions corresponding to steric repulsion are observed within the carbon frameworks. Overall, the RDG analysis complements the ELF and CDD results by identifying the noncovalent contributions in Al_13_–HBC and their secondary role in Al_13_–C_60_.

#### Charge Redistribution and Interfacial Electronic Structure

To quantify the magnitude and direction of charge transfer upon adsorption, fragment charges were analyzed using three complementary schemes: natural population analysis (NPA),^46^ Bader’s quantum theory of atoms in molecules (QTAIM),^47^ and atomic dipole moment corrected Hirshfeld (ADCH).^48^ Despite differences in partitioning schemes, ranging from orbital-based to real-space partitioning methods, all methods yield a consistent qualitative picture.

The NPA results (Table 2) reveal a clear contrast. In Al_13_–HBC, only a minimal net charge transfer of 0.16 |e| occurs from the HBC fragment to Al_13_. While this value is not negligible, it remains relatively small compared to near-integer charge transfer associated with complete electron transfer.^49,50^ This partial electronic redistribution indicates a polarization-dominated interaction, consistent with our charge-density difference, ELF, RDG, and PDOS analyses. HBC therefore behaves as a weak p-type ligand. In contrast, a substantial charge transfer of 1.18 |e| occurs from Al_13_ to C_60_, reflecting its n-type character. Together, these results distinguish the polarization-dominated physisorption in Al_13_–HBC from the charge-transfer-driven chemisorption in Al_13_–C_60_.

**Table 2:** Partial Charges of Al_13_ in Al_13_–HBC, Al_13_–C_60_, Solvated Al_13_HBC, and Al_13_–(HB-HBC) Obtained Using Natural Population Analysis, Quantum Theory of Atoms in Molecules (QTAIM), and Atomic Dipole Corrected Hirshfeld (ADCH) for the Different Complexes Considered^a^

	Al_13_–HBC	Al_13_–C_60_	Al_13_–HBC (sol)	Al_13_–(HB-HBC)
NPA	–0.16	1.18	–0.21	–0.17
QTAIM	0.05	1.41	0	0.05
ADCH	–0.38	0.21	–0.57	–0.42
Dipole moment (D)	–7.8	6.2	–13.8	–8.6

a The dipole moment is also reported, where a positive sign corresponds to Al_13_ as the positive pole.

A consistent picture is obtained from the real-space QTAIM analysis. The integrated charge over the Al_13_ atomic basins is nearly neutral (0.05 |e|) in Al_13_–HBC, indicating minimal ionic or covalent character. Conversely, Al_13_ in Al_13_–C_60_ carries a large positive charge (1.41 |e|), showing pronounced electron transfer accompanying bond formation. Although the large charge transfer indicates significant electron donation from Al_13_ to C_60_, redistributed electron density is not localized solely on the two fragments. Instead, the CDD plot reveals pronounced electron accumulation in the Al–C bonding region, while the ELF reveals pronounced high electron localization. These observations indicate that the transferred electron contributes directly to a covalent Al–C bond formation, rather than the interaction being adequately described as a purely ionic Al_13_^+^C_60_^–^ pair.

The partial charge obtained using ADCH further supports the opposite direction of the charge transfer. Al_13_ carries −0.38 |e| in Al_13_–HBC, whereas +0.21 |e| in Al_13_–C_60_. Importantly, the opposite signs indicate reversed dipole orientations, consistent with HBC acting as a donor and C_60_ as an acceptor.

These trends are reflected in the calculated dipole moments. Al_13_–HBC possesses a dipole moment of 7.8 D, with the positive pole on HBC, whereas Al_13_–C_60_ exhibits a dipole moment of 6.2 D, with the positive pole on Al_13_. The opposite orientations of the dipole moment confirm the reversed direction of interfacial polarization in the two systems.

While the absolute charge values depend on the population analysis methods, all approaches consistently predict opposite directions and markedly different magnitudes of charge transfer. These results reinforce the distinction between weak polarization-driven interaction in Al_13_–HBC and stronger charge-transfer interaction in Al_13_–C_60_. The resulting interfacial dipoles can also modify the local electrostatic environment, potentially influencing core-level binding energies. Importantly, a large dipole moment does not necessarily imply substantial charge transfer. In Al_13_–HBC, overlap between the occupied electron densities of the interacting fragments gives rise to Pauli pushback, in which exchange repulsion between occupied states displaces electron density away from the interface.^51^ This redistribution induces polarization within each fragment, shifting the centers of positive and negative charge and thereby producing a sizable interfacial dipole without requiring substantial interfragment charge transfer. Such adsorption-induced interface dipoles and complex charge-density redistribution have been reported for weakly interacting molecular interfaces.^52,53^ In contrast, the dipole in Al_13_–C_60_ is dominated by symmetry-allowed charge transfer and the accompanying electron redistribution resulting from strong frontier orbital hybridization.

An explicit energetic decomposition, such as absolutely localized molecular orbital energy decomposition analysis (ALMO-EDA), could in principle quantify energetic contributions from polarization and charge transfer.^54^ In the present work, we instead focus on the electronic structure of these interactions using charge partitioning, CDD, ELF, and PDOS analyses to characterize the interfacial electronic structure. These analyses consistently indicate that Al_13_–HBC is dominated by interfacial polarization with only minimal charge transfer, whereas Al_13_–C_60_ exhibits substantial charge transfer accompanied by strong orbital coupling and covalent bond formation.

This behavior is consistent with the XPS core-level shifts reported by Shibuta et al., which were interpreted as evidence of an electron-enriched Al_13_ on HB-HBC.^32,34^ However, such XPS shifts may also arise from interfacial polarization and do not uniquely imply substantial charge transfer.^35^ To directly simulate the experimental XPS response, we calculated core-level binding energies using a ΔSCF approach based on a penalty functional and the exact Coulomb cutoff method as implemented in OpenMX.^55^ As shown in Figure 3a, the Al 2*p* level in Al_13_–HBC shifts to lower binding energy relative to pristine Al_13_, whereas it shifts to higher binding energy in Al_13_–C_60_. Correspondingly (Figure 3b,c), the C 1*s* core level of HBC shifts to higher binding energy upon Al_13_ adsorption, while that of C_60_ shifts to lower binding energy. The calculated shifts reproduce the experimental trends and show quantitative agreement as shown in Table 3. For C 1*s*, the calculated shift upon Al_13_ adsorption is +0.25 eV for HBC, in excellent agreement with the experimental value of approximately +0.25 eV, while the corresponding shift for C_60_ is −0.60 eV compared with the experimental value of approximately −0.30 eV. The remaining deviation for Al_13_–C_60_ may partly reflect the different Al_13_:C_60_ stoichiometry between the computational model and the experimental system. Nevertheless and more importantly, the experimentally observed XPS signature of an electron-enriched Al_13_ on HBC is reproduced despite the minimal net charge transfer from HBC to Al_13_. This agreement therefore indicates that the observed core-level shift does not require substantial integer charge transfer and is instead predominantly associated with interfacial polarization.

**Figure 3:**
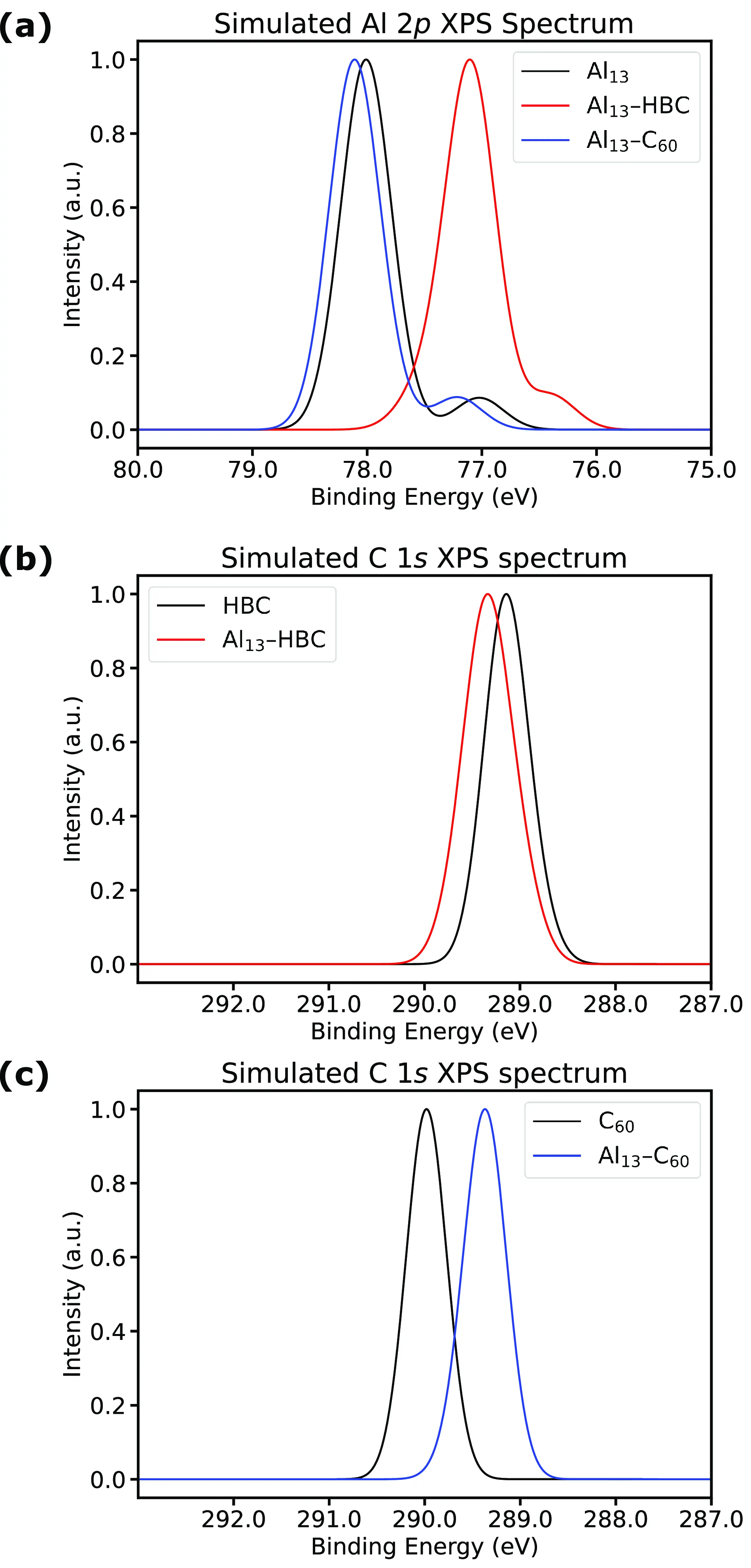
Simulated XPS spectrum as obtained from ΔSCF core-level shifts of (a) Al 2*p* and (b), (c) C 1*s* levels. A Gaussian broadening of FWHM = 0.5 eV is applied to all concerned states.

**Table 3:** Calculated and Experimental XPS Core-level Shifts upon Adsorption of Al_13_ on HBC and C_60_ ^a^

System	core level	ΔBE_calc._ (eV)	ΔBE_exp._ (eV)
Al_13_–HBC	C 1s	+0.25	≈+0.25
	Al 2p	–0.85	not available
Al_13_–C_60_	C 1s	–0.60	≈–0.30
	Al 2p	+0.10	not available

a The calculated shifts were obtained using the ΔSCF method.

Additional insight is provided by the spin-density distributions shown in Figure 4. In both complexes, the unpaired electron is predominantly localized on the Al_13_ fragment. In Al_13_–HBC, this localization indicates that Al_13_ largely retains its neutral, open-shell character upon adsorption, consistent with minimal net charge transfer. This weakly perturbed electronic state provides a possible explanation for the experimentally observed susceptibility of Al_13_ to oxidation on HBC upon exposure to O_2_. In contrast, in Al_13_–C_60_, the persistence of the unpaired electron on Al_13_ despite substantial net charge transfer implies that electron donation occurs primarily through paired electrons participating in Al–C bond formation, rather than through transfer of the unpaired electron. This electronic reorganization results in the oxidation of the Al_13_ cluster and an effective loss of its superatomic character already at the adsorption stage, in agreement with XPS measurements.

**Figure 4:**
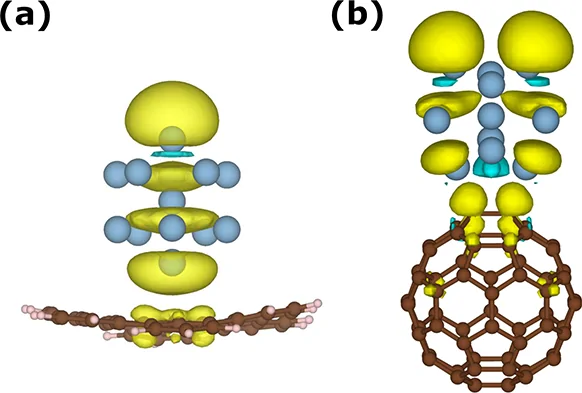
Spin density plots of (a) Al_13_–HBC and (b) Al_13_–C_60_.

#### Frontier Orbital Symmetry and Electronic Coupling

To rationalize the contrasting adsorption behaviors in terms of electronic structure, the energy alignment and symmetry compatibility of the frontier orbitals were analyzed using projected density of states (PDOS). The electronic structure of the Al_13_ cluster can be described within the jellium model, where its frontier states correspond to the 1F superatomic shell.^25^ The highest occupied and lowest unoccupied molecular orbitals of Al_13_ SA (HOMO_α_ and LUMO_β_) possess *A*_2*u*_ symmetry.^18^

Figure 5 compares the frontier orbital energies of the isolated fragments (red dotted lines) with their contributions after adsorption (red solid lines). The orbital energies of the isolated fragments are referenced to the vacuum level and therefore may appear above or below the Fermi level of the complex (black solid line). These isolated-fragment orbital energies are shown only as reference energies and should not be interpreted according to their position relative to the complex Fermi level.

**Figure 5:**
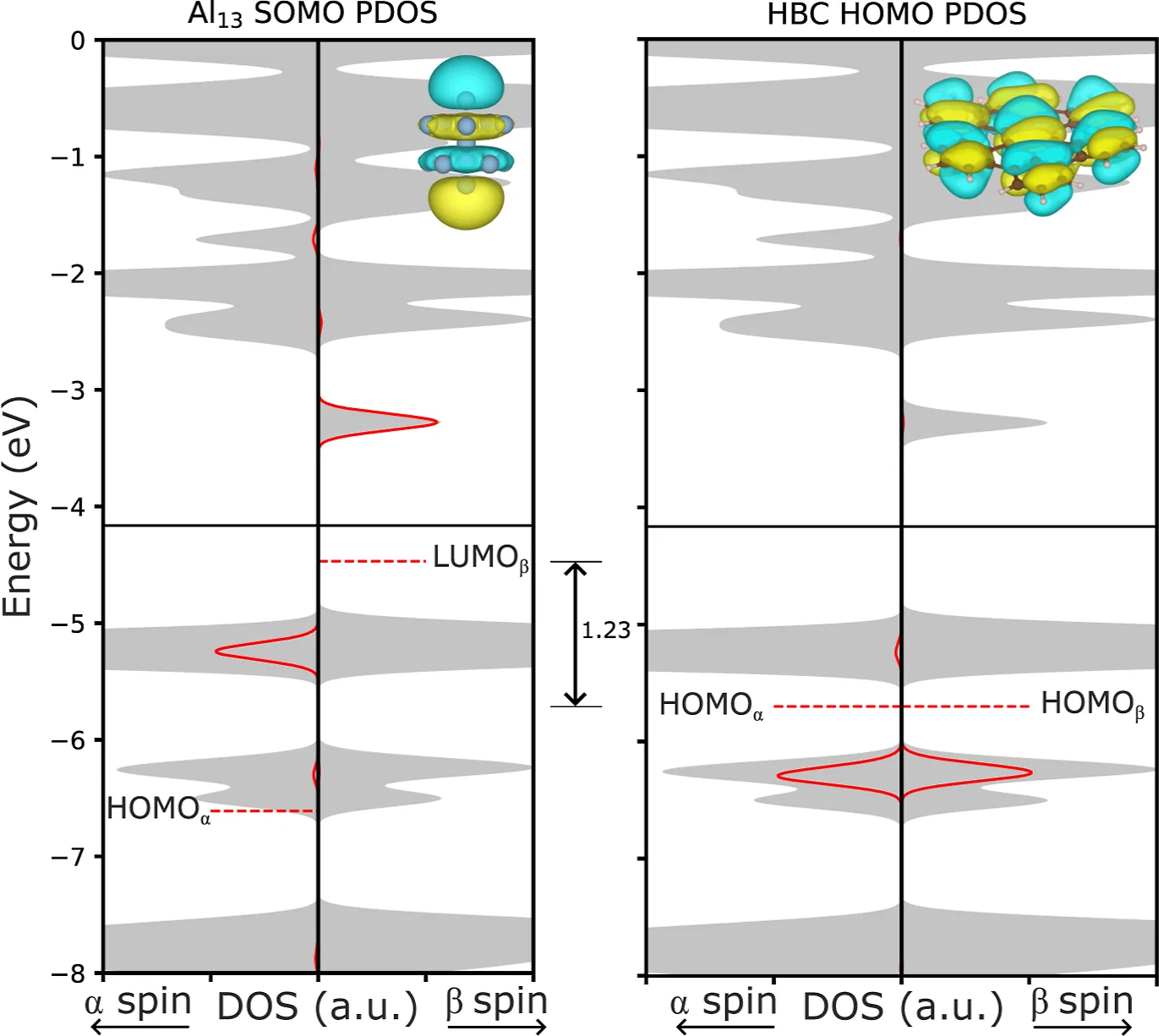
Spin-polarized projected density of states (PDOS) of the Al_13_–HBC interface. The energy scale is referenced to the vacuum level, and the black solid line indicates the Fermi level of the adsorbed complex. The gray background represents the total DOS. Red dotted lines indicate the energies of selected frontier orbitals of the isolated fragments before adsorption, while red solid lines represent their projected contribution to the electronic states of the adsorbed complex. Despite the small difference of the Al_13_ LUMO_β_ and HBC HOMO_β_ of 1.23 eV, both orbitals remain unhybridized indicating that the overlap is not favorable.

For Al_13_–HBC, the smallest frontier gap occurs between the HBC HOMO_β_ and the Al_13_ LUMO_β_, with an energy separation of 1.23 eV. Despite this relatively small gap, the PDOS of both orbitals after adsorption as shown in Figure 5 remain narrow and essentially unbroadened, indicating negligible hybridization. This is consistent with the small overlap integral, $\left|S_{12}\right|=\left|\left\langle \phi_{1}^{{Al}_{13}}|\phi_{2}^{HBC}\right\rangle \right|$ , between these orbitals, where $\phi_{1}^{{Al}_{13}}$ and ϕ_2_^HBC^ correspond to Al_13_ LUMO_β_ and HBC HOMO_β_, respectively, as shown in Figure 6, where the details of the mapping procedure are provided in the Supporting Information (Note S1). The overlap integral values remain uniformly small (maximum value ∼0.03). The spatial mapping of the overlap shows that the largest values are localized above the central carbon ring of HBC, and follow the π-type nodal pattern of the HBC HOMO_β_. This indicates that the adsorption geometry reflects the region of maximum frontier orbital overlap.

**Figure 6:**
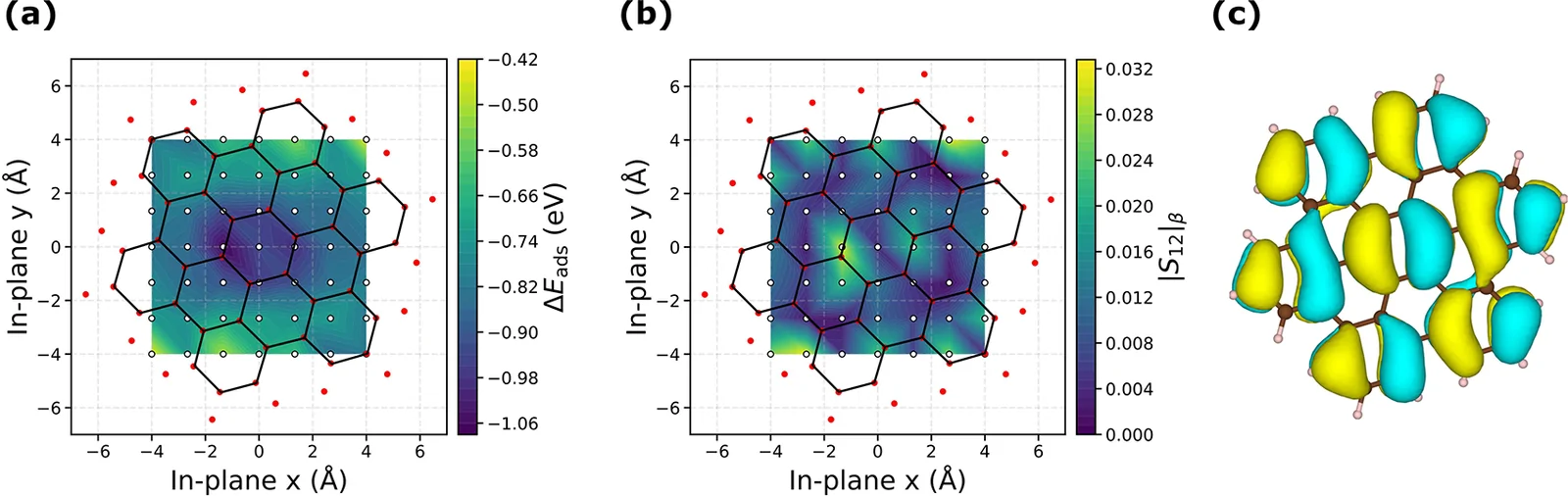
(a) Contour map of the adsorption energy (Δ*E*_ads_) for Al_13_ positioned above the HBC surface at fixed vertical separation. (b) Contour map of the magnitude of the overlap integral (|*S*_12_|) between the Al_13_ LUMO_β_ and the HBC HOMO_β_. (c) HOMO of HBC. The two maps display closely related spatial patterns, with the largest overlap and strongest adsorption occurring above the central aromatic ring region. The nodal structure observed in the map of the overlap integral reflects the symmetry of the HBC HOMO, indicating that the interaction strength is governed by the strong overlap and close spatial alignment of the interacting frontier orbitals.

However, the magnitude of the overlap remains intrinsically limited by symmetry. The HBC HOMO_β_ is characterized by delocalized π-type character with a nodal plane coincident with the central adsorption axis, whereas the Al_13_ LUMO_β_ exhibits predominantly σ-type symmetry centered along the adsorption axis, lacking such a nodal plane. The π–σ mismatch suppresses constructive overlap and efficient orbital mixing at high-symmetry sites. Consequently, Al_13_ undergoes a lateral displacement to align its σ density with the π lobes of the HBC. Despite this shift, the electronic coupling remains relatively weak, as evidenced by the negligible level broadening in the PDOS. The interaction is therefore symmetry-constrained and dominated primarily by dispersive contributions rather than by strong SA–molecule hybridization.

Alternatively, hybridization may occur with lower-lying HBC orbitals of σ character, which are symmetry-allowed for interaction with the Al_13_ LUMO_β_ state. However, such an orbital (σ-type symmetry HOMO_β_ – 8, Figure S2) lies 1.82 eV below the HOMO, resulting in a large effective donor–acceptor gap of 3.05 eV that renders such interaction energetically unfavorable. A similar situation holds in the α–spin channel, where the corresponding Al_13_ acceptor orbitals lie at even higher energy. Consequently, although polarization-driven charge redistribution can occur, the electronic structure does not favor the formation of a strongly coupled donor–acceptor pair capable of inducing full electron transfer or covalent bonding. This is consistent with the absence of significant orbital broadening or splitting in the PDOS.

In contrast, the interaction between Al_13_ and C_60_ satisfies both energetic and symmetry requirements for strong coupling. The dominant interaction arises between the Al_13_ HOMO_β_ – 1 and C_60_ LUMO, which possess compatible π-type character and transform according to the same *B*_2_ irreducible representation of *C*_2*v*_. The corresponding *b*_2_ orbitals are separated by only 1.77 eV. On the other hand, the Al_13_ HOMO_β_ contributes little to the interfacial interaction because its *B*_1_ symmetry is incompatible with the C_60_ acceptor orbital and remains essentially non-bonding. Instead, the HOMO_β_ – 1 provides the dominant contribution because it satisfies both energetic and symmetry requirements for efficient coupling. The overlap integral $\left|\left\langle \phi_{b_{2}}^{{Al}_{13}}|\phi_{b_{2}}^{C_{60}}\right\rangle \right|=0.13$ indicates substantial symmetry-allowed overlap. As shown in Figure 7, these orbitals exhibit pronounced broadening and hybridization upon adsorption, forming the HOMO of the complex. Although the donor and acceptor frontier orbitals of the isolated fragments are separated by approximately 1.77 eV, these orbital energies serve only as references prior to interaction. Upon adsorption, electronic relaxation and symmetry-allowed hybridization stabilize the interfacial electronic structure. This stabilization lowers the energy of the interacting system and provides the driving force for electron redistribution. Consequently, efficient charge transfer from Al_13_ to C_60_ occurs, accompanied by the formation of covalent Al–C bonds, as confirmed by the real-space analyses.

**Figure 7:**
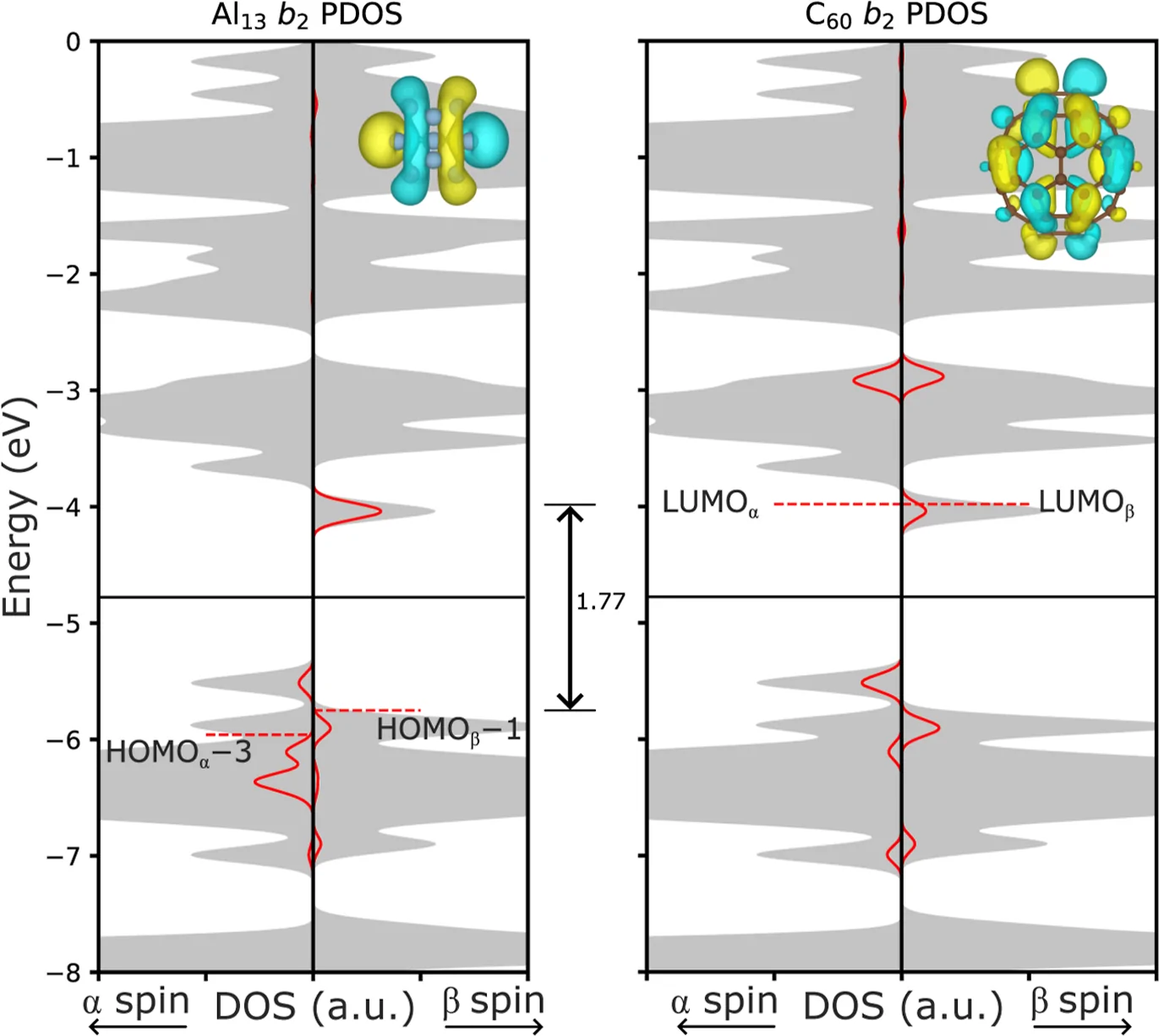
Projected density of states (PDOS) for Al_13_ HOMO_α_ – 3 (the same spatial component with HOMO_β_ – 1) and C_60_ LUMO, both have *b*_2_ symmetry. PDOS shows broadening as evidence of hybridization of the two orbitals. The energy of each state is computed relative to the vacuum. The black solid line marks the Fermi level of the Al_13_–C_60_ complex.

It is important to note that the present PDOS analysis does not aim to establish a general framework for multiligand coordination of Al_13_, where cooperative and multiorbital effects may become significant, nor to reproduce higher-order stoichiometries such as Al_13_– ${\left(C_{60}\right)}_{3}$ . Rather, it isolates the fundamental electronic factors governing charge transfer at the SA–organic interface. Taken together, these results indicate that favorable frontier orbital energy alignment alone is insufficient to promote electron transfer to or from Al_13_. Instead, both frontier orbital energy alignment and the magnitude of orbital overlap—ultimately dictated by symmetry—determine whether interfacial interactions lead to weak polarization (Al_13_–HBC) or strong chemisorption (Al_13_–C_60_).

To assess whether environmental effects can modify this behavior, we further examined the influence of environmental polarization and peripheral chemical substitution of the molecular framework (see Note 2 in the Supporting Information for details). Even under strongly polarizing conditions or upon electron-donating *tert*-butyl (HB) substitution, population analyses show no appreciable increase in charge transfer to Al_13_ (Table 2). This insensitivity to environmental and chemical perturbations reinforces that charge transfer is fundamentally governed by orbital energy alignment and symmetry, rather than by secondary environmental effects.

## Conclusion

In this work, we employed DFT to examine the electronic structure of Al_13_ at π-conjugated interfaces and to clarify the origin of experimentally observed XPS core-level shifts. In Al_13_–HBC, previous experiments reported core-level shifts consistent with an electron-enriched cluster and interpreted these as evidence of reduction to Al_13_^–^. Our calculations reproduce these XPS trends, but show that only minimal net charge transfer occurs. Instead, the observed core-level shifts arise predominantly from interfacial polarization. This resolves a key ambiguity in the interpretation of XPS at SA–organic interfaces, demonstrating that such signatures do not uniquely indicate integer charge transfer.

The electronic origin of this behavior is identified by contrasting Al_13_–HBC with Al_13_–C_60_. While energetic alignment between donor and acceptor states is necessary, efficient charge transfer occurs only when symmetry-allowed frontier orbital overlap enables strong electronic coupling. In Al_13_–HBC, the mismatch between the σ-type Al_13_ LUMO_β_ and the π-type HBC HOMO suppresses hybridization, leading to polarization-dominated interactions without substantial electron transfer. In contrast, symmetry-compatible orbitals in Al_13_–C_60_ enable strong coupling, resulting in charge transfer and covalent bond formation. Environmental perturbations enhance interfacial dipoles but do not alter this outcome, confirming that orbital symmetry, rather than external polarization, governs charge transfer in these systems.

Taken together, these results establish that XPS core-level shifts at SA–organic interfaces can arise predominantly from interfacial polarization, even in the absence of substantial integer electron transfer. More broadly, we show that charge transfer in superatomic systems is controlled not only by energetic alignment but critically by symmetry-allowed frontier orbital overlap. This provides a clear design principle: ligands must present frontier orbitals that are both energetically accessible and symmetry-compatible with superatomic states to enable efficient charge transfer. Such control opens a pathway toward stabilizing superatomic charge states, including Al_13_^–^, and toward the rational design of SA-based interfaces and functional materials.

## Supplementary Material



## Notes

Ground-state structure and higher-energy isomers of Al_13_–HBC (Figure S1), Ground-state structure and higher-energy isomers of Al_13_–C_60_ (Figure S2), RDG plots of Al_13_–HBC and Al_13_–C_60_ (Figure S3), fragment molecular orbital diagrams of Al_13_–HBC and Al_13_–C_60_ (Figure S4), construction of adsorption energy and overlap integral contour maps for Al_13_–HBC (Note S1), analysis of environmental polarization and substituent effects (Note S2), XYZ coordinates of HBC (Table S1), XYZ coordinates of C_60_ (Table S2), XYZ coordinates of Al_13_–HBC (Table S3), and XYZ coordinates of Al_13_–C_60_ (Table S4) (PDF)
