# Supplementary material for: Interfacial Polarization and Charge Transfer in Al13 Superatom–Organic Interfaces: Role of Frontier Orbital Overlap
Source: ACS Omega. 2026 Oct 6;11(42):64912–21. doi: 10.1021/acsomega.6c05879 (PMC13717776; doi:10.1021/acsomega.6c05879)
Supplement: Supplementary file 1 [file ao6c05879_si_001.pdf]

Supporting Information for Interfacial Polarization and Charge Transfer in Al_{13} Superatom–Organic Interfaces: Role of Frontier Orbital Overlap

Meliton R. Chiong III,† Atsushi Nakajima,*,‡ and Yoshitada Morikawa*,†,¶,§

†*Department of Precision Engineering, Graduate School of Engineering, The University of Osaka, 2-1 Yamadaoka, Suita, Osaka 565-0871, Japan*

‡*Department of Chemistry, Faculty of Science and Technology, Keio University, 3-14-1 Hiyoshi, Kohoku-ku, Yokohama 223-8522, Japan*

¶*Innovative Catalysis Science Division, Institute of Open and Transdisciplinary Research Initiatives (ICS-OTRI), The University of Osaka, Suita, Osaka 565-0871, Japan*

§*Research Center for Precision Engineering, Graduate School of Engineering, The University of Osaka, 2-1 Yamadaoka, Suita, Osaka 565-0871, Japan*

E-mail: nakajima.a7@keio.jp; morikawa@prec.eng.osaka-u.ac.jp

Phone: +81 (0)6 6879 7288. Fax: +81 (0)6 6879 7290

Contents

Figure S1: Ground-state structure and higher-energy isomers of Al_{13}–HBC S3

Figure S2: Ground-state structure and higher-energy isomers of Al_{13}–C_{60} S3

Figure S3: Reduced density gradient plots of Al_{13}–HBC and Al_{13}–C_{60} S4

Figure S4: Fragment molecular orbital diagram of Al_{13}–HBC and Al_{13}–C_{60} S5

Note S1: Construction of the Al_{13}–HBC Adsorption Energy and Overlap Integral Contour Maps S6

Note S2: Environmental Polarization and Substituent Effects S9

Table S1: XYZ coordinates of HBC S11

Table S2: XYZ coordinates of C_{60} S12

Table S3: XYZ coordinates of Al_{13}–HBC S13

Table S4: XYZ coordinates of Al_{13}–C_{60} S14

References S15

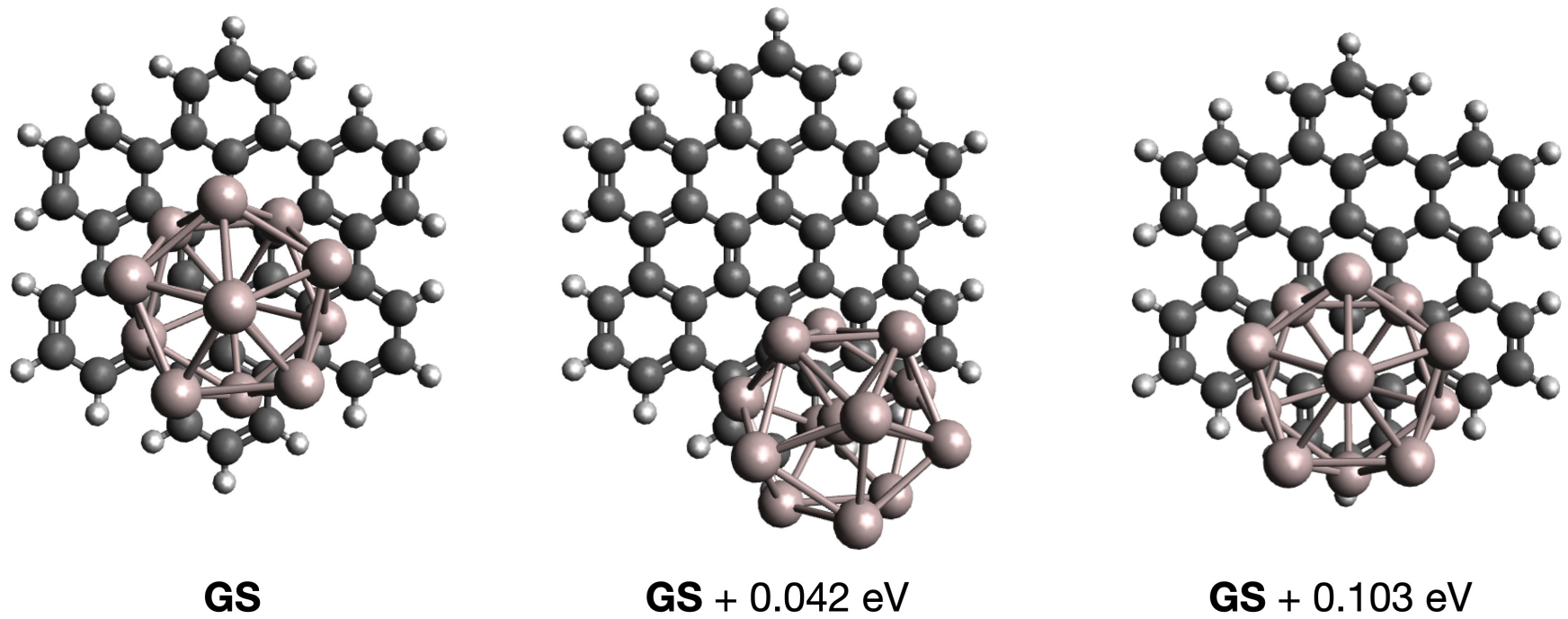

Figure S1: Ground-state structure and the two higher-energy isomers of Al_{13}–HBC obtained from the structural search. The relative energies are given with respect to the ground-state structure.

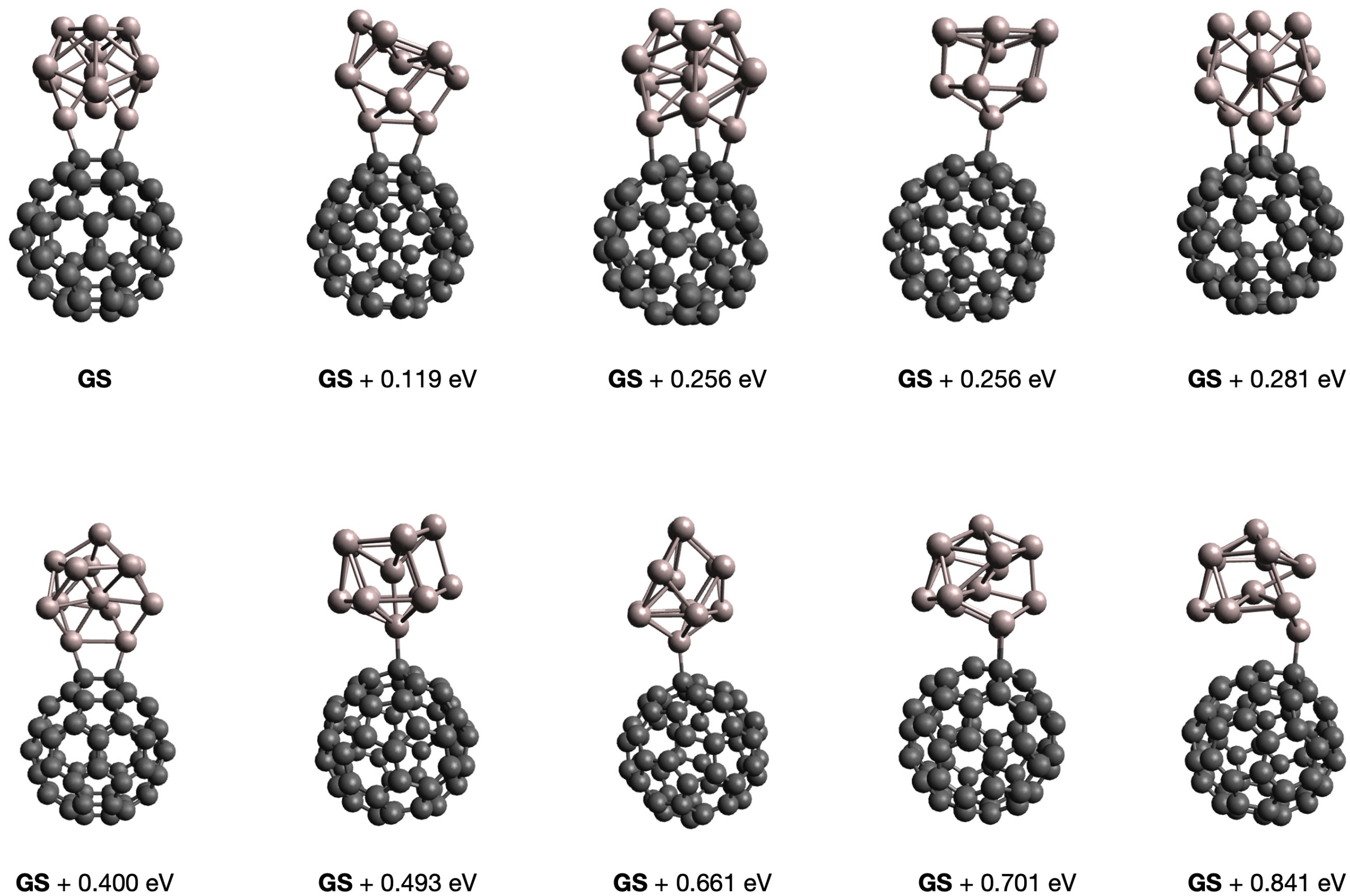

Figure S2: Ground-state structure and nine representative higher-energy isomers of Al_{13}–C_{60} obtained from the structural search. The relative energies are given with respect to the ground-state structure.

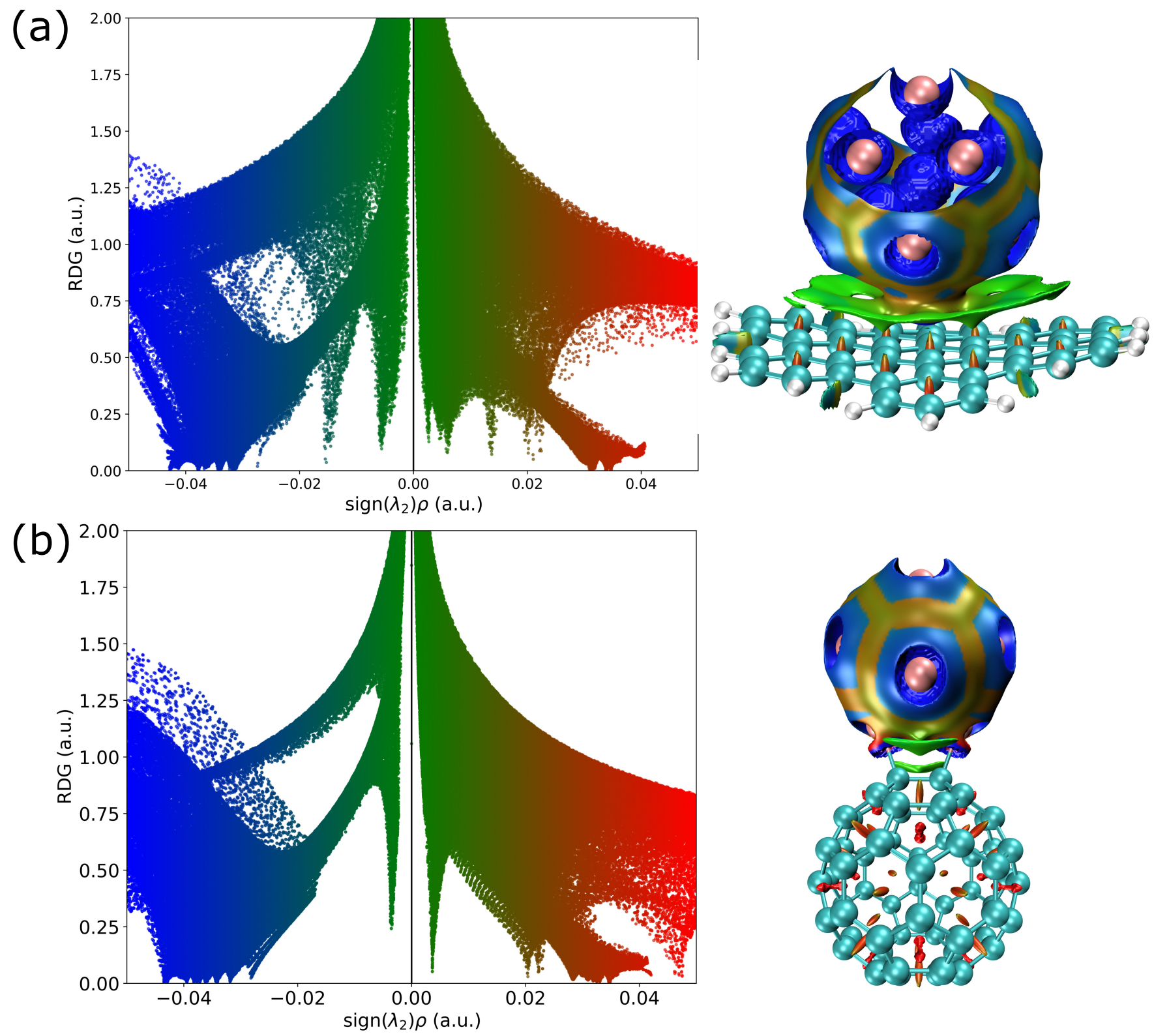

Figure S3: Reduced density gradient (RDG) plots of (a) Al_{13}–HBC and (b) Al_{13}–C_{60}. Green surfaces indicate regions of weak attractive van der Waals interactions. In Al_{13}–HBC, this features are localized at the interface, consistent with dispersion-dominated stabilization in the absence of covalent bonding.

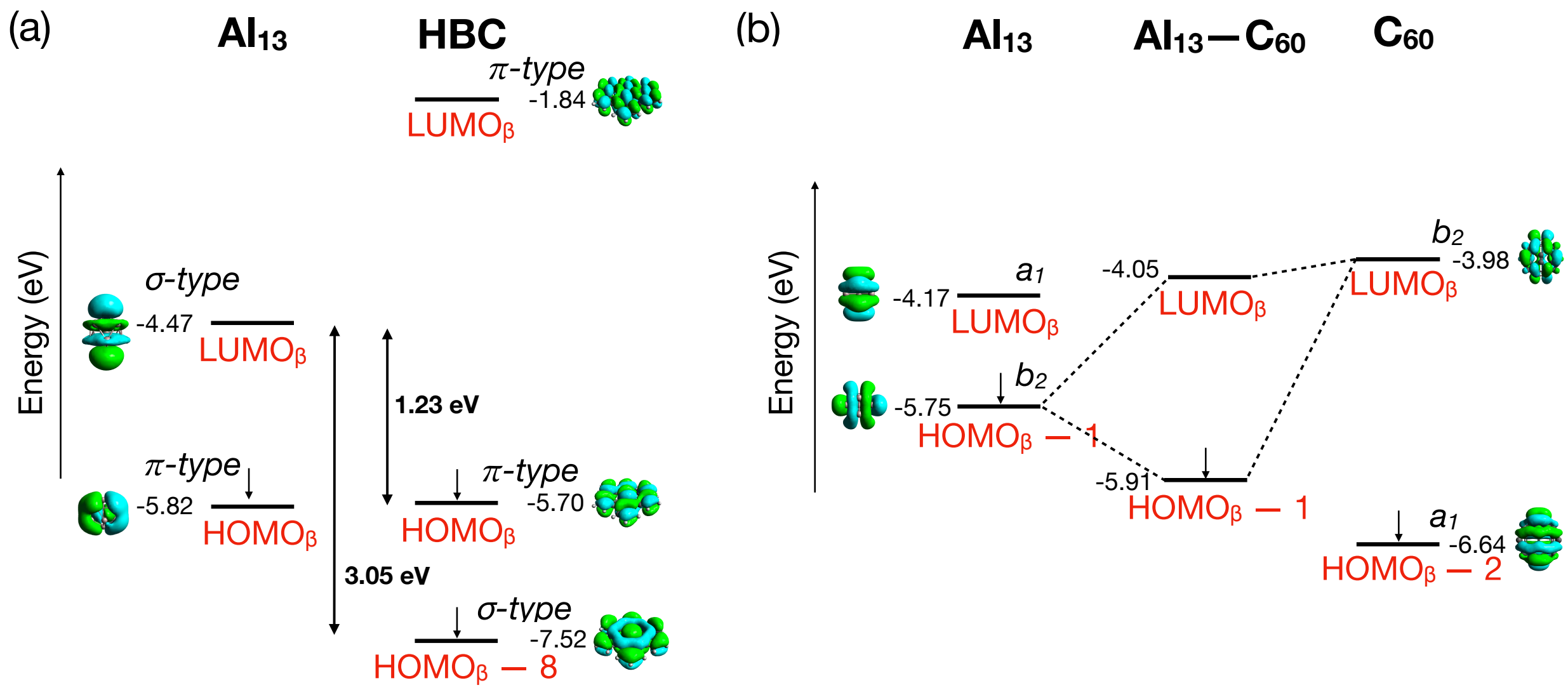

Figure S4: Frontier molecular orbital diagram (β spin channel) of (a) Al_{13}–HBC and (b) Al_{13}–C_{60}. Orbital energies are given in eV (grey numbers). In (a), the energy gap between the donor HBC HOMO$_\beta$ and the acceptor Al_{13} LUMO$_\beta$ is 1.23 eV. Despite this relatively small gap, electron transfer is disfavored due to poor orbital overlap arising from symmetry mismatch. Although HBC HOMO$_\beta - 8$ exhibits a nodal structure compatible with the Al_{13} LUMO$_\beta$, it lies 2.18 eV below the the HOMO$_\beta$ making too low in energy to contribute significantly to hybridization. In (b), the donor Al_{13} HOMO$_\beta$ $-$ 1 and the acceptor C_{60} LUMO are separated by 1.77 eV and share the same b_2 symmetry, enabling effective orbital overlap and facilitating charge transfer.

Note S1: Construction of the Al_{13}–HBC Adsorption Energy and Overlap Integral Contour Maps

To analyze the dependence of the Al_{13}–HBC interaction on the overlap integral of the Al_{13} cluster above the HBC surface, we constructed a two-dimensional interaction energy surface by translating the Al_{13} cluster parallel to the molecular plane of HBC while maintaining a fixed vertical separation.

Because HBC is not perfectly planar due to slight curvature and relaxation effects, a reference plane was first defined by a least-squares fit to the Cartesian coordinates of all HBC atoms. The molecular centroid was first computed as

$$\mathbf{r}_0 = \frac{1}{N}\sum_{i=1}^{N}\mathbf{r}_i, \tag{1}$$

and the coordinates were translated to the centroid frame, $\mathbf{x}_i = \mathbf{r}_i - \mathbf{r}_0$. The spatial covariance matrix

$$\mathbf{C} = \frac{1}{N}\sum_{i=1}^{N}\mathbf{x}_i\mathbf{x}_i^{\mathrm{T}} \tag{2}$$

was then diagonalized

$$\mathbf{C}\mathbf{v}_k = \lambda_k\mathbf{v}_k. \tag{3}$$

The eigenvector $\mathbf{v}_k$ associated with the smallest eigenvalue corresponds to the direction of minimal spatial variance and therefore defines the unit normal vector $\hat{\mathbf{n}}$ of the least-squares molecular plane. The remaining two eigenvectors correspond to directions of maximal variance within the molecular framework and form an orthonormal basis $\{\hat{\mathbf{e}}_1, \hat{\mathbf{e}}_2\}$ spanning the plane. These vectors satisfy

$$\hat{\mathbf{e}}_1 \cdot \hat{\mathbf{e}}_2 = 0, \qquad \hat{\mathbf{e}}_i \cdot \hat{\mathbf{n}} = 0. \tag{4}$$

This orthonormal coordinate system was used to project the positions of the Al_{13} cluster onto the HBC plane and to define the lateral coordinates employed in constructing the interaction energy surface. For each lateral grid point (x, y), a reference position within the molecular plane was defined as

$$\mathbf{r}_{\parallel}(x, y) = \mathbf{r}_0 + x\,\hat{\mathbf{e}}_1 + y\,\hat{\mathbf{e}}_2. \tag{5}$$

The Al_{13} cluster was then positioned such that a designated apex Al atom (the “tip” atom) was located at a fixed distance d above this plane along the surface normal:

$$\mathbf{r}_{\mathrm{tip}}(x, y) = \mathbf{r}_{\parallel}(x, y) + d\,\hat{\mathbf{n}}. \tag{6}$$

In this work, the vertical offset was fixed at $d = 2.51$ Å for all grid points.

Importantly, this construction enforces a constant separation measured perpendicular to the best-fit HBC plane rather than a constant distance to individual carbon atoms. As a consequence, the minimum Al–C distance varies modestly across the surface due to the intrinsic corrugation of the HBC framework. This approach provides a physically well-defined constant-height scan above the HBC surface, analogous to constant-height potential energy surface mappings commonly employed in surface science.

At each lateral (x, y) position, a single-point energy calculation was performed at the PBE0/6-311+G(d,p) level to evaluate the adsorption energy

$$\Delta E_{\mathrm{ads}} = E_{\mathrm{Al_{13}-HBC}} - E_{\mathrm{Al_{13}}} - E_{\mathrm{HBC}}, \tag{7}$$

where $E_{\mathrm{Al_{13}-HBC}}$ is the total energy of the combined system, and $E_{\mathrm{Al_{13}}}$ and E_{HBC} are the energies of the isolated Al_{13} cluster and HBC fragment, respectively. This procedure yields a two-dimensional potential energy surface describing the lateral dependence of the Al_{13}–HBC interaction at a fixed vertical separation.

To probe the electronic origin of the interaction, the magnitude of the overlap integral between the Al_{13} LUMO_β and the HBC HOMO_β was evaluated at each grid point,

$$|S_{12}|_\beta = \left|\left\langle \phi^{\mathrm{Al_{13}}}_{\mathrm{LUMO},\beta} \middle| \phi^{\mathrm{HBC}}_{\mathrm{HOMO}\beta} \right\rangle\right| . \tag{8}$$

The resulting discrete data points were interpolated using Delaunay triangulation to construct continuous contour maps. Figure 6 of the main text presents the resulting contour maps of the ΔE_{ads} and $|S_{12}|_\beta$. Both surfaces exhibit closely analogous spatial patterns. In particular, the central ring region of HBC shows larger $|S_{12}|_\beta$ values with clearly defined nodal structures. The spatial distribution closely resembles the shape of the HBC HOMO, indicating that the degree of interaction is governed by the orbital symmetry match between the Al_{13} singly occupied molecular orbital (SOMO) and the HBC HOMO. Consequently, regions exhibiting larger $|S_{12}|_\beta$ correspond to stronger adsorption interactions.

Note S2: Environmental Polarization and Substituent Effects

This section provides the details regarding the sensitivity of the Al_{13}–HBC complex to external polarization and chemical substitution, as summarized in the main text.

In the experimental system, Al_{13} is deposited on HB-HBC rather than pristine HBC. Adsorption of Al_{13} on HBC induces partial charge transfer and an interfacial dipole, raising the question of whether environmental polarization or chemical modification of the ligand can cooperatively enhance electron donation to the cluster.

To investigate the influence of environmental polarization, a polarizable continuum model was employed using a solvation model based on solute electron density (SMD) solvation scheme with water ($\varepsilon = 78.3553$).[1] This large dielectric constant was intentionally chosen to exaggerate polarization effects and test whether significant charge transfer can be induced under highly favorable conditions. As summarized in Table 2 of the main text, solvent polarization slightly increases the negative partial charge on Al_{13}, from -0.16 to -0.21 according to NPA. The ADCH analysis reflects this enhanced polarization more strongly. Correspondingly, the dipole moment of Al_{13}–HBC increases from 7.8 D to 13.8 D, with the positive pole on HBC. Despite this pronounced polarization, the net charge transfer remains small, particularly when evaluated using the real-space QTAIM analysis, which indicates negligible charge redistribution.

The effect of chemical substitution was examined by replacing the six vertex hydrogen atoms of HBC with *tert*-butyl (HB) groups to form HB-HBC. Experimentally, these bulky substituents serve to suppress intermolecular aggregation through steric repulsion, resulting in isolated HB-HBC molecules arranged in a flat, in-plane configuration on the surface.[2] In addition, HB groups are weakly electron donating and could enhance the donor character of the central π-system. However, as shown in Table 2, the effect of this substitution on the interaction with Al_{13} is minimal. The partial charges obtained from both NPA and QTAIM

remain close to those of the unsubstituted HBC, indicating that peripheral alkylation does not significantly modify the donor strength of the π-system. A small increase in the dipole moment, from 7.8 D to 8.6 D, is observed, reflected in a slightly more negative Al_{13} charge in the ADCH analysis.

Taken together, these results indicate that neither strong environmental polarization nor chemical substitution of HBC substantially alters the weak charge-transfer character in Al_{13}–HBC. Even under intentionally enhanced polarization conditions, electron donation to Al_{13} remains limited. In the present system, polarization alone is insufficient to induce significant charge transfer because the symmetry-adapted donor orbital capable of interacting with Al_{13} SOMO lies too low in energy to participate effectively.

XYZ coordinates of relevant structures

Table S1: XYZ coordinates of HBC (in Å).

Atom	x	y	z	Atom	x	y	z
C	-0.858539	-1.125096	-0.006397	C	0.545528	-1.305876	0.006220
C	-1.404166	0.180828	-0.004652	C	-1.730715	-2.269552	-0.071614
C	1.404175	-0.180748	0.004570	C	1.099333	-2.633936	0.071282
C	0.858310	1.124879	-0.015992	C	2.832976	-0.364859	-0.003222
C	-0.545361	1.305609	0.015848	C	1.730848	2.269703	-0.067059
C	-1.099422	2.634113	0.066768	C	-2.833002	0.364651	0.003584
C	-3.385491	1.667901	0.073818	C	-4.773614	1.820694	0.060070
C	-2.501085	2.821036	0.154190	C	-5.611881	0.723529	-0.006763
C	-5.079259	-0.550545	-0.069389	C	-3.697743	-0.755333	-0.077011
C	-3.134746	-2.095622	-0.148817	C	-3.951923	-3.219046	-0.294772
C	-3.414398	-4.491787	-0.344622	C	-2.048415	-4.670363	-0.227481
C	-1.190607	-3.579284	-0.073513	C	0.245040	-3.764141	0.072716
C	0.798468	-5.036978	0.226290	C	2.165125	-5.210300	0.343429
C	3.007539	-4.115217	0.294125	C	2.501621	-2.821361	0.148700
C	3.385789	-1.667337	0.077714	C	4.774148	-1.819068	0.071327
C	5.612144	-0.721427	0.009357	C	5.079142	0.552254	-0.058152
C	3.697594	0.755955	-0.073118	C	3.134147	2.095443	-0.154268
C	3.950620	3.218434	-0.307146	C	3.412644	4.490927	-0.360036
C	2.047143	4.669843	-0.237244	C	1.190108	3.579049	-0.077460
C	-0.244612	3.763785	0.076754	C	-0.797365	5.036149	0.236179
C	-2.163640	5.209022	0.358963	C	-3.006427	4.114294	0.306577
H	-5.216979	2.806924	0.095289	H	-6.687836	0.862364	-0.010358
H	-5.758548	-1.391567	-0.110076	H	-5.024418	-3.108946	-0.384683
H	-4.064593	-5.350230	-0.476055	H	-1.651610	-5.675941	-0.274990
H	0.159887	-5.909264	0.273426	H	2.576668	-6.205499	0.474444
H	4.072937	-4.280440	0.384013	H	5.218312	-2.804673	0.112635
H	6.688181	-0.859588	0.014053	H	5.757808	1.394085	-0.092780
H	5.022543	3.107682	-0.403300	H	4.062054	5.348836	-0.498674
H	1.649429	5.674941	-0.288266	H	-0.158024	5.907737	0.286867
H	-2.574558	6.203502	0.497218	H	-4.071435	4.278745	0.402722

Table S2: XYZ coordinates of C_{60} (in Å).

Atom	x	y	z	Atom	x	y	z
C	2.221882	0.591137	2.680661	C	3.125198	0.159810	1.637349
C	1.338710	-0.314854	3.252148	C	3.109337	-1.160305	1.207550
C	3.207693	-1.462479	-0.203004	C	3.240303	1.232344	0.674484
C	3.334779	0.942055	-0.679980	C	3.318258	-0.432601	-1.127527
C	-0.448872	1.366421	3.225672	C	0.470709	2.310143	2.629995
C	-0.023667	0.080588	3.531296	C	1.779539	1.930699	2.363469
C	2.408828	2.326934	1.123128	C	-0.262539	3.101557	1.667584
C	0.341629	3.482318	0.477040	C	1.704369	3.086985	0.199099
C	-2.132240	-0.849099	2.684628	C	-2.574659	0.490062	2.366098
C	-0.882373	-1.049574	3.254972	C	-1.750425	1.575152	2.631499
C	-1.635030	2.647359	1.668544	C	-3.318258	0.432601	1.127527
C	-3.207693	1.462479	0.203004	C	-2.349110	2.592521	0.479221
C	-0.501372	-2.993864	1.805671	C	-1.803173	-2.785105	1.211710
C	-0.050223	-2.143574	2.806297	C	-2.601925	-1.734324	1.642308
C	-3.334779	-0.942055	0.679980	C	-1.704369	-3.086985	-0.199099
C	-2.408828	-2.326934	-1.123128	C	-3.240303	-1.232344	-0.674484
C	2.189832	-2.103938	1.803034	C	1.719924	-2.989087	0.760721
C	1.322509	-1.689664	2.804869	C	0.401843	-3.424849	0.761861
C	-0.341629	-3.482318	-0.477040	C	2.349110	-2.592521	-0.479221
C	1.635030	-2.647359	-1.668544	C	0.262539	-3.101557	-1.667584
C	-2.221882	-0.591137	-2.680661	C	-1.779539	-1.930699	-2.363469
C	-0.470709	-2.310143	-2.629995	C	-1.338710	0.314854	-3.252148
C	-2.189832	2.103938	-1.803034	C	-3.109337	1.160305	-1.207550
C	-3.125198	-0.159810	-1.637349	C	-1.322509	1.689664	-2.804869
C	0.501372	2.993864	-1.805671	C	-0.401843	3.424849	-0.761861
C	-1.719924	2.989087	-0.760721	C	0.050223	2.143574	-2.806297
C	2.132240	0.849099	-2.684628	C	2.601925	1.734324	-1.642308
C	1.803173	2.785105	-1.211710	C	0.882373	1.049574	-3.254972
C	0.448872	-1.366421	-3.225672	C	1.750425	-1.575152	-2.631499
C	2.574659	-0.490062	-2.366098	C	0.023667	-0.080588	-3.531296

Table S3: XYZ coordinates of Al_{13}–HBC (in Å).

Atom	x	y	z	Atom	x	y	z
C	2.735394	-0.149733	0.205991	C	2.299047	1.144993	0.581016
C	2.101700	-1.299928	0.736616	C	3.814538	-0.297135	-0.732406
C	1.254164	1.295538	1.520750	C	2.896080	2.310537	-0.013579
C	0.622969	0.140039	2.072573	C	0.755138	2.601849	1.855295
C	1.071817	-1.162265	1.697406	C	-0.351710	0.288999	3.134584
C	0.430451	-2.323847	2.254976	C	2.460747	-2.609006	0.262469
C	1.757789	-3.751214	0.718373	C	2.042985	-4.993084	0.148262
C	0.756967	-3.611384	1.765853	C	3.008253	-5.122989	-0.833676
C	3.726060	-4.017591	-1.251763	C	3.479934	-2.755125	-0.708846
C	4.234186	-1.588100	-1.137393	C	5.364793	-1.713125	-1.946342
C	6.053561	-0.595842	-2.382019	C	5.610482	0.669396	-2.042096
C	4.486676	0.845391	-1.232291	C	3.984286	2.172497	-0.909709
C	4.535890	3.317358	-1.488103	C	4.044559	4.574716	-1.188996
C	2.982718	4.714985	-0.314612	C	2.385999	3.600355	0.277325
C	1.263629	3.743356	1.191935	C	0.661734	4.982298	1.424582
C	-0.397989	5.106198	2.303771	C	-0.861037	4.001232	2.996660
C	-0.287310	2.744626	2.803866	C	-0.765144	1.580096	3.534201
C	-1.632848	1.705967	4.619416	C	-2.115937	0.587194	5.274324
C	-1.758285	-0.678893	4.846655	C	-0.890506	-0.854549	3.767247
C	-0.545038	-2.180589	3.273037	C	-1.189042	-3.319636	3.756227
C	-0.865492	-4.576778	3.277967	C	0.101661	-4.722491	2.301014
H	1.496892	-5.875336	0.454455	H	3.198327	-6.093012	-1.280331
H	4.466767	-4.142915	-2.030705	H	5.729925	-2.692228	-2.227842
H	6.940515	-0.711642	-2.995723	H	6.162367	1.525298	-2.407357
H	5.353591	3.236647	-2.191931	H	4.489679	5.452421	-1.645198
H	2.623511	5.711191	-0.093169	H	1.001544	5.864195	0.898054
H	-0.871241	6.071642	2.447040	H	-1.700750	4.120439	3.668866
H	-1.924559	2.686094	4.973832	H	-2.778096	0.702650	6.125665
H	-2.153954	-1.535780	5.375684	H	-1.967939	-3.233717	4.502142
H	-1.377151	-5.450824	3.666378	H	0.337803	-5.719068	1.952716
Al	-0.928497	0.013902	0.105403	Al	-0.414480	-1.408949	-2.195139
Al	-2.376278	-2.264772	-0.392347	Al	-0.401078	1.401434	-2.203921
Al	-3.587934	0.013938	0.726816	Al	-2.880448	-2.282172	-3.120194
Al	-2.650311	0.007212	-1.784112	Al	-2.371145	2.286718	-0.404646
Al	-1.646025	-0.004694	-4.243467	Al	-4.848949	-1.404173	-1.294544
Al	-2.858708	2.292010	-3.130130	Al	-4.843483	1.431461	-1.299725
Al	-4.393467	0.014861	-3.665220				

Table S4: XYZ coordinates of Al_{13}–C_{60} (in Å).

Atom	x	y	z	Atom	x	y	z
C	2.735394	-0.149733	0.205991	C	2.299047	1.144993	0.581016
C	2.101700	-1.299928	0.736616	C	3.814538	-0.297135	-0.732406
C	1.254164	1.295538	1.520750	C	2.896080	2.310537	-0.013579
C	0.622969	0.140039	2.072573	C	0.755138	2.601849	1.855295
C	1.071817	-1.162265	1.697406	C	-0.351710	0.288999	3.134584
C	0.430451	-2.323847	2.254976	C	2.460747	-2.609006	0.262469
C	1.757789	-3.751214	0.718373	C	2.042985	-4.993084	0.148262
C	0.756967	-3.611384	1.765853	C	3.008253	-5.122989	-0.833676
C	3.726060	-4.017591	-1.251763	C	3.479934	-2.755125	-0.708846
C	4.234186	-1.588100	-1.137393	C	5.364793	-1.713125	-1.946342
C	6.053561	-0.595842	-2.382019	C	5.610482	0.669396	-2.042096
C	4.486676	0.845391	-1.232291	C	3.984286	2.172497	-0.909709
C	4.535890	3.317358	-1.488103	C	4.044559	4.574716	-1.188996
C	2.982718	4.714985	-0.314612	C	2.385999	3.600355	0.277325
C	1.263629	3.743356	1.191935	C	0.661734	4.982298	1.424582
C	-0.397989	5.106198	2.303771	C	-0.861037	4.001232	2.996660
C	-0.287310	2.744626	2.803866	C	-0.765144	1.580096	3.534201
C	-1.632848	1.705967	4.619416	C	-2.115937	0.587194	5.274324
C	-1.758285	-0.678893	4.846655	C	-0.890506	-0.854549	3.767247
C	-0.545038	-2.180589	3.273037	C	-1.189042	-3.319636	3.756227
C	-0.865492	-4.576778	3.277967	C	0.101661	-4.722491	2.301014
H	1.496892	-5.875336	0.454455	H	3.198327	-6.093012	-1.280331
H	4.466767	-4.142915	-2.030705	H	5.729925	-2.692228	-2.227842
H	6.940515	-0.711642	-2.995723	H	6.162367	1.525298	-2.407357
H	5.353591	3.236647	-2.191931	H	4.489679	5.452421	-1.645198
H	2.623511	5.711191	-0.093169	H	1.001544	5.864195	0.898054
H	-0.871241	6.071642	2.447040	H	-1.700750	4.120439	3.668866
H	-1.924559	2.686094	4.973832	H	-2.778096	0.702650	6.125665
H	-2.153954	-1.535780	5.375684	H	-1.967939	-3.233717	4.502142
H	-1.377151	-5.450824	3.666378	H	0.337803	-5.719068	1.952716
Al	-0.928497	0.013902	0.105403	Al	-0.414480	-1.408949	-2.195139
Al	-2.376278	-2.264772	-0.392347	Al	-0.401078	1.401434	-2.203921
Al	-3.587934	0.013938	0.726816	Al	-2.880448	-2.282172	-3.120194
Al	-2.650311	0.007212	-1.784112	Al	-2.371145	2.286718	-0.404646
Al	-1.646025	-0.004694	-4.243467	Al	-4.848949	-1.404173	-1.294544
Al	-2.858708	2.292010	-3.130130	Al	-4.843483	1.431461	-1.299725
Al	-4.393467	0.014861	-3.665220				

References

(1) Marenich, A. V.; Cramer, C. J.; Truhlar, D. G. Universal solvation model based on solute electron density and a continuum model of the solven defined by the bulk dielectric constant and atomic surface tensions. *J. Phys. Chem. B* **2009**, *113*, 6378–6396.

(2) Motojima, J.; Suzuki, N.; Tsukuda, H.; Yokoyama, T. Adsorption and self-assembly of hexa-*tert*-butyl-hexa-*peri*-hexabenzocoronene on Si(111)-$\sqrt{3} \times \sqrt{3}$-Ag surface. *Surf. Sci.* **2021**, *713*, 121905.
